# Effects of Tillage and Nitrogen Fertilizers on CH_4_ and CO_2_ Emissions and Soil Organic Carbon in Paddy Fields of Central China

**DOI:** 10.1371/journal.pone.0034642

**Published:** 2012-05-04

**Authors:** Li Cheng-Fang, Zhou Dan-Na, Kou Zhi-Kui, Zhang Zhi-Sheng, Wang Jin-Ping, Cai Ming-Li, Cao Cou-Gui

**Affiliations:** 1 College of Plant Science and Technology, Huazhong Agricultural University, Wuhan, Hubei, China; 2 Institute of Animal Husbandry and Veterinary Science, Hubei Academy of Aguicultural Sciences, Wuhan, Hubei, China; USDA-ARS, United States of America

## Abstract

Quantifying carbon (C) sequestration in paddy soils is necessary to help better understand the effect of agricultural practices on the C cycle. The objective of the present study was to assess the effects of tillage practices [conventional tillage (CT) and no-tillage (NT)] and the application of nitrogen (N) fertilizer (0 and 210 kg N ha^−1^) on fluxes of CH_4_ and CO_2_, and soil organic C (SOC) sequestration during the 2009 and 2010 rice growing seasons in central China. Application of N fertilizer significantly increased CH_4_ emissions by 13%–66% and SOC by 21%–94% irrespective of soil sampling depths, but had no effect on CO_2_ emissions in either year. Tillage significantly affected CH_4_ and CO_2_ emissions, where NT significantly decreased CH_4_ emissions by 10%–36% but increased CO_2_ emissions by 22%–40% in both years. The effects of tillage on the SOC varied with the depth of soil sampling. NT significantly increased the SOC by 7%–48% in the 0–5 cm layer compared with CT. However, there was no significant difference in the SOC between NT and CT across the entire 0–20 cm layer. Hence, our results suggest that the potential of SOC sequestration in NT paddy fields may be overestimated in central China if only surface soil samples are considered.

## Introduction

Global surface temperatures have increased by 0.88°C since the late nineteenth century [1]. The observed climate changes are caused by the emission of greenhouse gases (GHGs) mainly through anthropogenic activities. Methane and CO_2_ are the most important GHGs, respectively contributing 15% and 60% to the anthropogenic GHG effect [2]. Rice paddies are an important source of atmospheric CH_4_. The amount of CH_4_ emitted from wetland paddy fields accounts for 10% to 20% of the total CH_4_ emissions (i.e. 50 Tg yr^−1^ to 100 Tg yr^−1^) [2]. The rice production of China exceeds that of any other country, accounting for 30% of the world total [3]. Agricultural activity affects CH_4_ and CO_2_ emissions, contributing 39% of the excess CH_4_ and 1% of the excess CO_2_ to global emissions [4]. Hence, CH_4_ emissions from paddy fields under different agricultural management practices in China are relevant to the discussion of the global C cycle and climate changes.

The entire process of CH_4_ emission from rice fields, including production, oxidation, and transport into the atmosphere is influenced by agricultural management practices, such as tillage and N fertilizer use [5]–[7]. Tillage affects a range of biological, chemical, and physical properties, thereby affecting the release of CH_4_ [8]. No-tillage (NT) has been reported to reduce CH_4_ emissions from paddy soils because rice straw is placed on the soil surface under NT and the soil conditions are more oxidative than those of conventional tillage (CT) [7], [9]. CH_4_ emissions from paddy fields are reportedly affected by the form and amount of N fertilizer applied [10]. Overall, the effects of N fertilizer application on CH_4_ fluxes from paddy fields are mostly unclear. Therefore, more research on the effects of N addition on CH_4_ emissions is needed.

Tillage practices can affect soil biochemical and physical properties, consequently influencing the release of CO_2_ [8]. However, there is no consensus on the differences in the soil CO_2_ emissions between NT- and CT-treated paddy fields. Some authors have reported similar soil CO_2_ fluxes from NT- and CT-treated paddy fields [7]. However, Liang et al. [9] reported higher soil CO_2_ emissions from CT-treated paddy fields than from the NT paddy fields. Nitrogen supplied by commercial fertilizers can be expected to affect soil CO_2_ flux by increasing the C input from enhanced plant productivity and crop residues returned to the soil [11]. However, studies on the effects of N fertilizer on soil CO_2_ emissions reveal diverse results [12]. Within the past few years, Iqbal et al. [13] and Xiao et al. [14] observed increased CO_2_ emissions from paddy soils because of a positive effect of N fertilization on plant biomass. However, Burton et al. [15] and DeForest et al. [16] found that the use of N reduced extracellular enzymatic activities and fungal populations, resulting in decreased soil CO_2_ flux. The effect of N fertilization on variation in CO_2_ emission under anaerobic conditions in paddy soils remains unknown.

Land management practices are increasingly thought to affect soil carbon levels and may partially ameliorate CO_2_ emissions and climate change [17], [18]. Studies have indicated that NT can increase C sequestration in paddy soils compared with CT [19]–[21]. In 2007, Tang et al. [20] indicated that the NT could sequester 112.3 kg C ha^−1^ yr^−1^ in the top 20 cm of purple paddy soil in the Beipei district of Chongqing City, China. In a 12-year study, Gao et al. [21] reported that NT could sequester 26.68 kg C ha^−1^ yr^−1^ in gray fluvoaguic paddy soils to a depth of 30 cm in Zhangjiagang City, Jiangsu Province, China. However, Six et al. [22] and Su [23] indicated that the effects of NT on SOC sequestration depend on the soil type. In a 5-year study, He et al. [24] indicated that NT did not increase the SOC sequestration of paddy fields in the 20 cm layer of sandy silty loam in Ningxiang country, Hunan Province. However, Angers and Eriksen-Hamel [25] reviewed the related literature and concluded that soil variables do not affect the tillage effects on soil C sequestration. Hence, further research is needed to clarify the effects of soil type on C sequestration in NT-treated soils.

No-tillage may influence SOC accumulation when soil surface layers are considered, but the effect may not be detected more deeply [22]. The influence of NT on SOC sequestration is still unclear. Hence, Baker et al. [26] analyzed sampling strategies on the potential of SOC sequestration under conservation tillage and indicated that SOC sequestration under this tillage varied with soil depth. Thus, shallow sampling may not be sufficient to assess the differences in SOC sequestration between NT- and CT-treated soils, and further research on the effects of deeper soil sampling on SOC sequestration in NT-treated soils should be performed.

Application of N fertilizer may play a significant role in the soil C sequestration [17]. Application of N fertilizer affects the soil C stock in two ways. These compounds can increase the crop biomass and influence the microbial decomposition of crop residues by affecting the N availability [27]. However, a meta-analysis of 111 studies covering 12 soil types of divergent ecosystems indicated that the effects of N fertilizer application on soil C content vary with the soil type although N fertilizer application consistently increases the crop biomass [28]. For example, Tong et al. [29] found in a 17-year study published in 2009 that the use of chemical N fertilizers did not increase the SOC content in a hydromorphic paddy soil in Hunan Province compared with no fertilizer use. By contrast, Shang et al. [30] found in the same province that increased N fertilization increased the SOC sequestration in paddy soils derived from quaternary red clay.

Central China is one of the major rice-producing regions in the country, comprising 28% of the total area cultivated with rice in China [31]. Recently, NT practices have become increasingly popular in this region. However, to our knowledge, relatively few studies have been performed on the effects of tillage and N fertilizer on CH_4_ and CO_2_ emissions as well as on SOC sequestration in the paddy fields in this region. We hypothesized that tillage practices and N fertilizer use affect CH_4_ and CO_2_ emissions as well as soil C sequestration in hydromorphic paddy fields in this region. We specifically tested the effects of tillage practices and N fertilizer use on SOC in soils from 0 cm to 5 cm, as well as from 0 cm to 20 cm, during the 2009 and 2010 rice growing seasons. This paper also aimed to evaluate the effects of tillage and N fertilizer on CH_4_ and CO_2_ emissions during the rice growing seasons.

## Results

### Temperature

The air temperature in the experimental site is shown in Table 1. The mean monthly air temperature ranged from 21.4°C to 28.9°C and from 19.7°C to 29.8°C during the 2009 and 2010 rice growing season, respectively. The mean monthly air temperature during rice growing seasons in 2009 was slightly lower than that in 2010. The mean air temperature from June to September, except for August, was significantly higher (*P*<0.05) in 2010 than in 2009.

**Table 1:** Mean monthly air temperature during rice growing season in the experimental site/°C.

Time	2009	2010
June	26.1 b	27.1 a
July	28.9 a	29.1 a
August	28.0 b	28.8 a
September	24.7 b	25.6 a
October	21.4 a	19.7 a
Mean air temperature during the ricegrowing season	26.7 a	27.4 a

Different letters in a line mean significant differences at the 5% level.

### CH_4_ and CO_2_ Emissions

**Figure 1:**
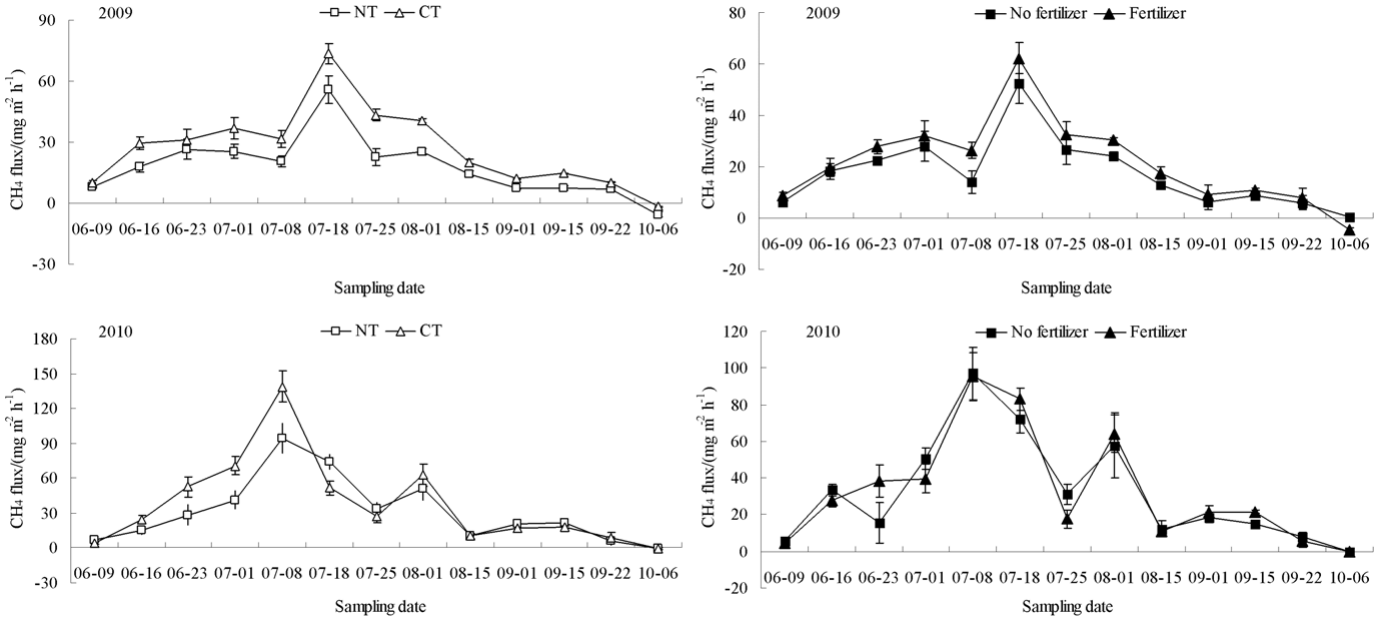
Changes in CH_4_ emission fluxes from paddy fields under different management practices during the 2009 and 2010 rice growing seasons. The vertical bars are standard deviations of the mean, n = 3.

The pattern of seasonal CH_4_ emission fluxes was similar across NT and CT treatments during the 2009 and 2010 rice growing seasons (Fig. 1). In both years, the CH_4_ emission fluxes in the four treatment groups were all initially low, increased gradually, and then peaked in mid-July (about 4–5 weeks after sowing). Thereafter, the CH_4_ emission fluxes declined gradually and remained relatively low until harvesting when the CH_4_ emission fluxes were lowest.

Application of N fertilizer significantly increased CH_4_ emissions by 13%–66% in 2009 and 2010 (*P*<0.05) (Table 2). Tillage significantly affected CH_4_ emissions, where NT significantly decreased CH_4_ emissions by 10%–36% compared with CT (*P*<0.05). No significant effect of tillage×fertilizer on the cumulative CH_4_ emissions was observed in 2009 or 2010. The cumulative CH_4_ emissions in 2010 were 1.39–2.45 times those recorded in 2009.

**Table 2:** Cumulative CH_4_ and CO_2_ emissions (g m^−2^) from different tillage treatments in the 2009 and 2010 rice growing seasons, n = 3.

Tillage	N fertilizer	Cumulative CH_4_ emissions		Cumulative CO_2_ emissions	
		2009	2010	2009	2010
NT	No fertilizer	2.74(0.57)	6.72(0.91)	125.7(10.6)	326.1(15.6)
	Fertilizer	4.54(0.44)	7.56(1.02)	140.1(6.6)	386.8(10.5)
CT	No fertilizer	4.28(0.27)	7.49(0.33)	103.4(7.2)	252.8(12.2)
	Fertilizer	6.76(0.40)	9.40(0.60)	100.3(4.3)	293.8(14.1)
Analysis of variance					
T		*	*	**	*
F		*	*	NS	NS
T×F		NS	NS	*	NS

T, tillage;

F, application of N fertilizer;

* , significant at the 0.05 probability level;

** , significant at the 0.01 probability level;

NS, not significant;

The values in brackets are standard deviations of the mean.

**Figure 2:**
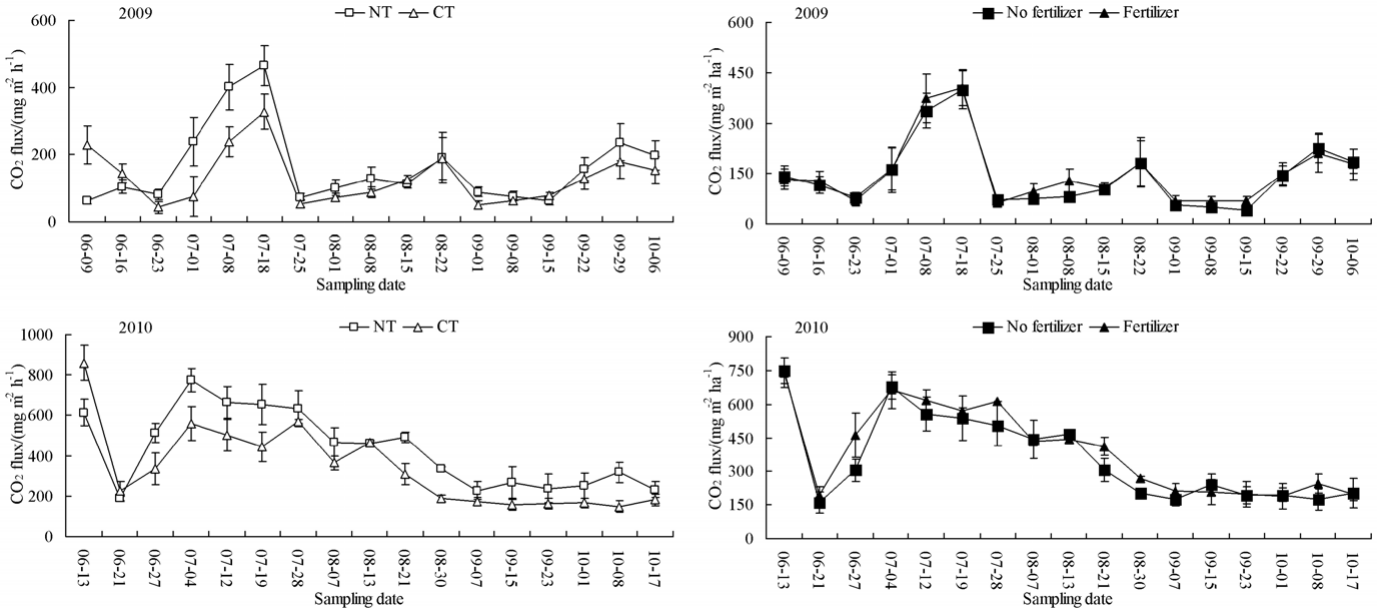
Changes in CO_2_ emission fluxes from paddy fields under different management practices during the 2009 and 2010 rice growing seasons. The vertical bars are standard deviations of the mean, n = 3.

Tillage treatments exhibited clear seasonal variations in soil CO_2_ fluxes in the 2009 and 2010 rice growing seasons (Fig. 2). The soil CO_2_ fluxes remained relatively low for the first two weeks after tillage, increased rapidly, stayed relatively high until about the middle 10 days of July, and then decreased to relatively low levels. Just one day after tillage (June 9, 2009 and June 13, 2010), the soil CO_2_ fluxes from CT were 1.40–4.60 times higher than those from NT (*P*<0.05).

The cumulative CO_2_ emissions from NT were 1.30–1.33 times those of CT (*P*<0.05) (Table 2). The application of N fertilizer had no significant effect on cumulative CO_2_ emissions. We observed a significant effect of tillage×fertilizer on CO_2_ emissions in 2009 (*P*<0.05) but not in 2010. In addition, cumulative CO_2_ emissions in 2010 were 2.44–2.93 times those in 2009.

### Soil Organic C and Bulk Density

As shown in Table 3, neither tillage nor N fertilizer application had any significant effect on bulk density before tilling fields or at harvesting in either year irrespective of the soil sampling depth. The SOC contents were significantly higher at 0–5 cm depth than at 0–20 cm depth under NT. In both years, application of N fertilizer significantly increased the SOC content by 4%–9% at harvesting and the SOC at the end of the growing seasons in 2009 and 2010 (21–94%) irrespective of soil sampling depths. Though NT had slightly higher SOC at the end of the growing seasons at 0–20 cm depth than CT in 2009 and 2010, we observed no significant effect of tillage or tillage×fertilizer in either year. However, across both years, tillage affected the SOC at 0–5 cm depth at harvesting, where NT significantly increased SOC contents by 12%–15% and SOC sequestration by 102%–270% than CT.

**Table 3:** SOC contents (g kg^−1^) and bulk density (g cm^−3^) before tillage and at harvesting, and SOC sequestration (kg C ha^−1^) based on soil sampling depths from different tillage treatments in the 2009 and 2010 rice growing seasons, n = 3.

Tillage	N fertilizer	2009									
		0–20 cm					0–5 cm				
		Bulk density before tillage	Bulk densityat harvesting	SOC contents before tillage	SOC contentsat harvesting	SOCsequestration	Bulk density before tillage	Bulk densityat harvesting	SOC contents before tillage	SOC contentsat harvesting	SOC sequestration
NT	No fertilizer	1.19 (0.02)	1.19 (0.01)	18.40 (0.78)	20.16 b (1.12)	2318 (129)	1.20 (0.02)	1.22 (0.03)	18.92 (1.06)	22.55 a (1.22)	1389 (115)
	Fertilizer	1.19 (0.05)	1.17 (0.03)	18.89 (0.91)	21.77 b(1.00)	3439 (271)	1.21 (0.04)	1.23 (0.02)	19.47 (1.23)	23.90 a (4.15)	1685 (162)
CT	No fertilizer	1.17 (0.03)	1.18 (0.04)	18.10 (0.85)	19.70 a (0.89)	2187 (148)	1.18 (0.03)	1.20 (0.06)	18.32 (1.73)	19.68 a (2.11)	559 (90)
	Fertilizer	1.18 (0.04)	1.20 (0.05)	18.56 (1.02)	20.59 a (1.14)	3146 (347)	1.19 (0.05)	1.19 (0.06)	18.85 (1.15)	20.81 a (2.94)	835 (117)
Analysis of variance											
T		–	NS	–	NS	NS	–	NS	–	**	**
F		–	NS	–	*	*	–	NS	–	*	**
T×F		–	NS	–	NS	NS	–	NS	–	NS	NS
**Tillage**	**N fertilizer**	**2010**									
		**0–20 cm**					**0–5 cm**				
		**Bulk density before tillage**	**Bulk density** **at harvesting**	**SOC contents before tillage**	**SOC contents at** **harvesting**	**SOC** **sequestration**	**Bulk density** **before tillage**	**Bulk density at** **harvesting**	**SOC contents** **before tillage**	**SOC contents** **at harvesting**	**SOC sequestration**
NT	No fertilizer	1.19 (0.04)	1.25 (0.07)	18.76 (0.88)	19.18 b (1.21)	2102 (123)	1.19 (0.03)	1.25 (0.06)	19.96 (1.25)	21.92 a (1.42)	1032 (66)
	Fertilizer	1.18 (0.04)	1.25 (0.04)	18.85 (1.06)	19.81 b (1.44)	3630 (310)	1.21 (0.04)	1.23 (0.08)	19.93 (1.25)	23.87 a (1.64)	1492 (122)
CT	No fertilizer	1.18 (0.06)	1.24 (0.06)	18.63 (0.76)	18.81 a (1.35)	1949 (251)	1.19 (0.04)	1.21 (0.07)	19.05 (0.97)	19.53 a (1.54)	279 (57)
	Fertilizer	1.17 (0.03)	1.27 (0.04)	18.80 (1.11)	19.33 b (1.51)	2877 (346)	1.20 (0.05)	1.21 (0.07)	19.70 (1.33)	21.15 a (1.71)	542 (115)
Analysis of variance											
T		–	NS	–	NS	NS	–	NS	–	*	**
F		–	NS	–	*	**	–	NS	–	**	**
T×F		–	NS	–	NS	NS	–	NS	–	NS	NS

T, tillage; F, application of N fertilizer;

* , significant at the 0.05 probability level;

** , significant at the 0.01 probability level; NS, not significant; SOC, soil organic C.

Different letters in a year at different depths mean significant differences at the 5% level.

The values in brackets are standard deviations of the mean.

Based on the SOC content and bulk density at harvesting in the plow layer (0–20 cm; Table 3), we estimated SOC at harvesting in the plow layer to be 27.0–29.5 t C ha^−1^ in 2009 and 2010. Correspondingly, annual SOC accumulation rate in the plow layer was estimated to be 0.06–0.14 t C ha^−1^ yr^−1^ for no fertilizer treatments and 0.25–0.47 t C ha^−1^ yr^−1^ for fertilizer treatments, with an average of 0.23 t C ha^−1^ yr^−1^ over the period 2009–2010.

## Discussion

### CH_4_ Emission

Application of N fertilizer in the present study increased CH_4_ emissions from paddy fields because of the promotion of rice growth, providing additional C sources and emission pathways [32]. Lindau and Bollich [33], in a study on a Louisiana rice field, which also had a humid subtropical climate, reported similar results from silt loam soil. However, Wassmann et al. [34] and Lu et al. [35] indicated no significant effect of N fertilizer application on CH_4_ emissions from paddy fields in Zhejiang Province, China. Schütz et al. [36] found that the application of urea significantly decreased CH_4_ emissions from paddy fields in Italy. Results varied among studies because of the differences in soil texture or climate. These findings show that further study is needed to understand the functioning of these complex and dynamic systems.

No-tillage significantly decreased CH_4_ emissions relative to CT in the present study. This is in accordance with the findings reported by Harada et al. [7] and Liang et al. [9]. The decrease in CH_4_ emissions under NT may be attributed to the differences regarding the size and activity of the methanotrophic community between tillage treatments [37]. Tillage also affects gaseous diffusivity and the rate of supply of atmospheric CH_4_ [38]. By contrast, NT improves macroporosity and maintains its continuity [39]. The improvement probably allows greater air diffusion, increasing CH_4_ uptake and decreasing CH_4_ emissions.

### CO_2_ Emissions

Application of N fertilizer increases plant biomass production, stimulating soil biological activity, and consequently, CO_2_ emission [40]. Wilson and Al-Kaisi [41], as well as Iqbal et al. [13], observed increased CO_2_ emissions caused by N fertilizer application. By contrast, Burton et al. [15] and DeForest et al. [16] indicated that reduced extracellular enzyme activities and fungal populations resulting from N fertilizer application resulted in decreased soil CO_2_ emissions. We observed no significant effect of N fertilizer application on cumulative CO_2_ emissions (Table 2), consistent with the results reported by Almaraz et al. [42]. This finding may be due to the fact that CO_2_ is reduced to CH_4_ under anaerobic conditions, thus leading to significant differences in CH_4_ emissions rather than in CO_2_ emissions between fertilized and unfertilized treatment areas (see Table 2).

We observed greater CO_2_ emissions from NT than from CT during the 2009 and 2010 rice growing seasons (Table 2). Similar results were obtained by Liu et al. [43] and Oorts et al. [8]. The differences between the soil CO_2_ emissions under the tillage treatments may have been caused by variation in soil C mineralization. Our own previously published work and those of other researchers indicated greater soil C mineralization under NT [7], [8], [44]. Increased SOC (Table 3) and higher microbial activity on the soil surface under NT [39] also resulted in greater soil CO_2_ emissions for NT than CT. However, CT is generally reported to increase CO_2_ emissions by exposing organic matter to more oxidizing conditions of the topsoil and accelerating the decomposition of aggregate-associated soil organic matter [38], [45]. The increased levels of surface crop residues in NT probably serve as a barrier for CO_2_ emissions from soil, decreasing the decomposition of crop residues because of reduced soil temperature and minimum soil-residue contact [46]. The inconsistent tillage effects on soil CO_2_ fluxes suggest that tillage is not the only factor affecting CO_2_ flux and that other factors are also involved. As suggested by Mosier et al. [47], CO_2_ emissions caused by NT may be similar or slightly lower than those caused by CT if entire growing and fallow seasons are considered.

We observed a significant effect of tillage×N fertilizer on cumulative CO_2_ emissions in 2009, in accordance with the results reported by Roberson et al. [48]. The cumulative CO_2_ emissions during the rice growing seasons in the present study were 1003–1401 kg C ha^−1^ in 2009 and 2528–3868 kg C ha^−1^ in 2010. These values were greater than 363–371 and 506–926 kg C ha^−1^ of cumulative CO_2_ emissions from different rice tillage systems at an Ogata farm (Japan) and the Hailun Experimental Station of Ecology (Heilongjiang Province, China), respectively [7], [9]. The differences in the emissions are possibly related to the dissimilar climates. The experimental field (humid mid-subtropical monsoon climate) in the present study is located at a lower latitude than those of the aforementioned studies.

We observed only one peak of CH_4_ or CO_2_ emission at the complete tillering stage, in contrast to the two or three emission peaks observed by other researchers [7], [31], [32]. The discrepancies are likely related to the different rice cropping systems (e.g. single, early, or late rice cropping), field pre-cropping management (e.g. rape and wheat), soil properties, weather conditions, and the use of N fertilizer [31]. The peak of CH_4_ emission in the present study may be attributed to (1) the higher availability of substrates through root exudation or decayed plant residues for methanogenic bacteria in the rice rhizosphere [49], [50] and (2) vigorous respiration by rice plants during this stage [51]. These processes promote CH_4_ emission because most of the CH_4_ is emitted through plants [52]. The peak of soil CO_2_ emission might be attributed to the increased availability of substrates from root exudation or microbial decomposition of left-over plant residues at the active vegetative growth stage.

Higher cumulative CH_4_ and CO_2_ emissions (Table 2) were observed during the rice growing season in 2010 than in 2009. Similar interannual differences between CH_4_ and CO_2_ emissions have been found by other researchers [31], [53]. Although these interannual differences in emissions are difficult to explain, discrepancies in climatic conditions and pre-crop residue management are probably involved. Residues of rapeseed were burnt before the experiment was started in 2009, which may be an important reason for the significantly lower emissions observed in this year. Higher mean air temperatures from June to August in 2010 than 2009 may be another important factor that led to higher cumulative CH_4_ and CO_2_ emissions.

### Soil Organic C

In the present study, N fertilizer application had a positive effect on SOC (Table 3). This is attributed to more rice biomass and in turn more residue input to soil under the N fertilized treatments [19]. Others [19], [30], [54], [55] also reported similar results. However, there were other reports indicating that application of chemical N fertilizers caused no significant or even negative effects on SOC [56]–[59]. The inconsistent results might depend on differences in the climatic and soil conditions, crop residue management, tillage regime, and experimental duration [60].

Here topsoil SOC (27.0–29.5 t C ha^−1^) was comparable to the results of Pan et al. (27.9–30.9 t C ha^−1^) [61], but lower than previous estimates of SOC of double-rice paddy soils reported by Shang et al. (36.4–48.2 t C ha^−1^) [30] and Wang et al. (32.7–41.9 t C ha^−1^) [62]. The SOC accumulation rate averaged 0.23 t C ha^−1^ yr^−1^ over the period 2009–2010 in the present study, generally lower than previous estimates in some double-rice paddy soils under short- or long-term chemical N fertilizer application [30], [61], [62]. However, it falls within the SOC sequestration rate range of 0.13–2.20 t C ha^−1^ yr^−1^ estimated by Pan et al. [63]. Lower levels of SOC in the present study could be attributed to differences in crop rotation systems. The decomposition rate of SOC in the single rice paddy-upland rotation system was higher than double rice-cropping paddy soils primarily dominated by surface waterlogging [30].

NT significantly increased SOC contents relative to CT at 0–5 cm depth but not at 0–20 cm depths (Table 3). A possible reason could be the return of moderately higher residues and root biomass to the soil surface, instead of migrating deeper into the soil under NT. CT incorporates residues into a greater soil volume [64], [65], resulting in relatively high SOC contents at deeper depths than NT [44]. Consequently, the lower SOC content at deeper depths under NT may weaken the tillage effects on SOC contents in the 20 cm layer. Similar observations were reported by other researchers [66], [67].

The present results indicate that tillage has different effects on SOC sequestration based on the soil sampling depth (Table 3). NT significantly increased SOC compared with CT only at 0–5 cm but not at 0–20 cm. This result is likely caused by the residue accumulation on the soil surface. Similar results were observed by Wright et al. [65] and Wright and Hons [68]. Our results were in contrast to the results reported by other researchers [69]–[71]. Nyamadzawo et al. [69] found that NT had more SOC than CT at 0–20 cm depth. Deen and Kataki [70] reported that, compared to CT, NT increased SOC storage only for the surface layer (0–5 cm) but had significantly lower SOC for the entire soil profile (0–40 cm). However, Christopher et al. [71] found that NT had similar amounts of SOC to CT across the entire soil profile (0–60 cm). We can speculate that the potential of SOC sequestration under NT paddy fields in the present study may be overestimated at deeper soil depths (>20 cm). Further research is needed to understand the sequestration of SOC under CT and NT systems based on different soil sampling depths.

## Materials and Methods

### Site Description

The experimental site is situated at an experimental farm in Zhonggui Country, Dafashi Town, Wuxue City, Hubei Province, China (29°55′ N, 115°30′ E). This region has a humid mid-subtropical monsoon climate, an average annual temperature of 16.8°C, and a mean annual precipitation of 1360.6 mm. Rainfall mostly occurred between April and August in the past 5 years. The paddy field soil is a hydromorphic paddy soil, which is silty clay loam (3% sand, 50% silt, and 47% clay) derived from quaternary yellow sediment. The main soil properties (0–20 cm depth) of the site are as follows: pH (extracted by H_2_O; soil: water = 1∶2.5), 6.58; organic C, 18.29 g kg^−1^; total N, 1.05 g kg^−1^; NO_3_^−^–N, 4.37 mg kg^−1^; NH_4_^+^–N, 2.43 mg kg^−1^; total P, 0.70 g kg^−1^; Bray-P, 3.65 mg kg^−1^; and available K (extracted by CH_3_COONH_4_), 111 mg kg^−1^.

The rice variety planted was *Liangyoupeijiu* (*Oryza sativa* L.), a mid-season rice variety. The experimental site was cultivated with a rape (*Brassica napus*)–rice (*Oryza sativa* L.) rotation. Rice was directly seeded from May to October each year and rape was planted from October to May the following year for the past 30 years.

### Experimental Design

Implementation of NT was initiated in 2006. Treatments were established following a split-plot design of a randomized complete block with standard tillage practices in the main plot and N fertilizers in the sub-plots. Each treatment had three replications. Each plot was isolated with a plastic film driven to a depth of 40 cm along the inner edge of the field ridge (30 cm at the base and 30 cm in height) in order to prevent lateral water movement caused by either leakage or permeable lateral flow. Each plot had an area of 45 m^2^ and an inlet for irrigation as well as an outlet for drainage. Two water meters were installed at the inlet and outlet to record water flow.

The weeds were controlled by spraying 36% glyphosate at 3 L ha^−1^ on June 4, 2009 and June 10, 2010. The field was then flooded on June 5, 2009 and June 12, 2010, respectively. Thereafter, the CT treatments were cultivated to 8–10 cm depth by hoeing, and were subsequently mouldboard ploughed twice to 20 cm depth before sowing. There was no tillage in the NT-treated subplots. Before sowing, rice seeds were soaked in water for 12 h and mixed with Dry-Raised Nurse (provided by Yangzhou Lvyuan Biochemial Co., LTD), a biological seed coat agent that can promote rice seed germination at a ratio of 1∶3 ratio. Rice seeds were sown manually at a rate of 22.5 kg ha^−1^ on June 8, 2009 and June 12, 2010. The crops were then harvested on October 8, 2009 and October 17, 2010, respectively. Commercial inorganic N–phosphorus (P)–potassium (K) fertilizer (15% N, 15% P_2_O_5_, 15% K_2_O), urea (46% N), single superphosphate (16% P_2_O_5_) and potassium chloride (60% K_2_O) were used to furnish 210 kg N ha^−1^, 135 kg P_2_O_5_ ha^−1^ and 240 kg K_2_O ha^−1^ during the rice growing season. Nitrogen fertilizers were broadcast at a rate of 84 kg N ha^−1^ as basal fertilizers immediately after sowing. The P and K fertilizers were only used as basal fertilizers immediately after seeding. The remaining N fertilizers were split into three doses of 42 kg N ha^−1^ on June 24, July 19 and August 12, 2009, as well as June 25, July 21, and August 14, 2010. The irrigation and application of pesticide were the same in all experimental treatments. According to local conventional irrigation-drainage practices, the plots were irrigated immediately upon the germination of rice seeds. Thereafter, the plots were reirrigated to a depth of 10 cm whenever that the water depth decreased to 1 cm to 2 cm above the soil surface during the growing season. The fields were not flooded for the entire 2 weeks before the rice was harvested.

### Methane Emission

Closed steel cylinders with diameters of 58 cm and height of 110 cm were used to quantify the CH_4_ fluxes from all plots during the rice growing seasons [72]. CH_4_ gas samples were collected from June 9 to October 8, 2009 and from June 12 to October 17, 2010. Two permanent rings were placed below water level to create a seal in each treatment plot and chambers were temporarily placed on these rings to measure the gas fluxes. Fans installed on the tops of the chambers were run for 1 min to mix the air within the chamber before each gas sample was taken. Then the gases in the chamber were drawn off with a syringe and immediately transferred into a 20 ml vacuum glass container. Three gas samples from the chamber headspace were collected at 8 min intervals using 25 ml plastic syringes during a half-hour period. Measurements of CH_4_ fluxes were conducted twice a day in the morning (9∶00 to 11∶00) and afternoon (15∶00 to17∶00). The morning and afternoon measurements from each plot were then averaged and considered as representative of that plot. The gas samples were collected 1 day after each N fertilizer application, and weekly.

We measured CH_4_ concentrations with gas chromatograph meter (Shimadzu GC-14B), fitted with a 6′ to 1/8′ stainless steel column (Porapack N, length×inner diameter: 3 m×2 mm) and a flame ionization detector as previously presented [73]. For determination of CH_4_, N_2_ (flow rate: 330 ml min^−1^), H_2_ (flow rate: 30 ml min^−1^) and zero air (flow rate: 400 ml min^−1^) were used as the carrier, fuel, and supporting gas, respectively. The temperatures of the column, injector, and detector were set at 55, 100, and 200°C, respectively. The changes in CH_4_ concentrations remained linear throughout the sampling period. The gas emission flux was calculated from the difference in the gas concentration according to the equation given by Zheng et al. [74]:
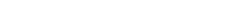
where *F* is the gas emission flux (mg m^−2^ h^−1^), *ρ* is the gas density at the standard state, *h* is the height of the chamber above the soil (m), *C* is the gas mixing ratio concentration (mg m^−3^), and *T* is the mean air temperature inside the chamber during sampling.

### Carbon Dioxide Emission

The soil CO_2_ flux was measured using the soil respiration method described by Parkinson [75]. In this method, a cylinder static chamber of 20 cm in diameter and 30 cm in height was placed on the soil and the rate of increase in CO_2_ concentration within the chamber was monitored using a LI–6400 portable photosynthesis analyzer (Li–Cor Inc., Lincoln, NE). We measured soil fluxes from 2 h measurements between 9∶00 and 11∶00 (a representative time of daily averages in this region described by Lou et al. [76]). The soil CO_2_ flux in the present study was measured in this way. The soil CO_2_ fluxes were measured 17 times at weekly intervals from June 9 to October 6, 2009, and 17 times at a 7–10 day interval from June 13 to October 17, 2010.

Each soil CO_2_ flux was determined every 1 min for 20 min. Three measurements were performed for each plot on each sampling day, and soil CO_2_ flux was the average of three individual measurements. Meteorological data were collected from the weather station in Wuxue City, 1 km from the experimental site.

Changes in the concentration with the sampling time were used to calculate the soil CO_2_ flux rate. The flux rate was calculated by simple linear regression when the concentration of gas inside the chamber varied linearly over time. Otherwise, the rate flux was calculated by nonlinear regression [77]. For the nonlinear regression, a model based on Fick’s law was fitted to the chamber data:
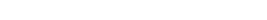
where the regression parameter *C*_0_ is the air concentration at time *t* = 0; *C*_max_ is the maximum concentration that can be reached in the chamber, and *k* is a rate constant. The values of *C*_max_, *C*_0_ and *k* were estimated iteratively using the observed concentration versus time data. Methane and CO_2_ fluxes were both expressed as mg m^−2^ h^−1^.

The cumulative CH_4_ and CO_2_ emissions were calculated for each plot by linearly interpolating the gas emissions between sampling dates under the assumption that the measured fluxes represented the average daily fluxes. The cumulative emissions were calculated according to the following equation:
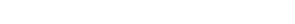
where *CE* is the cumulative emissions (g m^−2^), *F_i_* and *F_i+1_* are the measured fluxes of two consecutive sampling days (mg m^−2^ h^−1^), and *t* is the number of days between two consecutive sampling days (d).

### Sampling and Analytical Methods

Paddy soil samples (0–5 or 0–20 cm depth) were collected using a soil sampler with a diameter of 5 cm at eight random positions in each plot 1 day before the field was tilled and immediately after rice was harvested. The SOC were determined by dichromate oxidation and titration with ferrous ammonium sulfate [78]. The soil bulk density was determined by the method as described by Bao [78]. Soil bulk density samples for 0–5 or 0–20 cm soil layers were collected from each plot using metallic cores of 5.3 cm in diameter and 5.0 cm tall or 5.3 cm in diameter and 20 cm tall. Three soil cores were collected from each plot at 0–5 cm depth. The soil bulk density was computed as the weight to volume ratio of oven-dried (105°C) soil. Each measurement was replicated thrice. The SOC density (kg C ha^−1^) at the soil depth was evaluated by the methods described by Lu et al. [19]. The SOC density was calculated as follows:
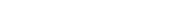
where *SOCD* and *SOCC* are the SOC density (kg C ha^−1^) and SOC concentration (g kg^−1^), respectively; *BD* is the soil bulk density, and *H* is the soil sampling depth in the paddy field.

### Statistical Analysis of Data

The SPSS 16.0 analytical software package was used for all statistical analyses. All data (mean±SE, *n* = 3) were checked for normal distribution. Statistical analysis was performed by two–way ANOVA to analyze the effects of N fertilizer and tillage on the CH_4_ and CO_2_ flux, as well as other C indices, using the SPSS general linear model procedure. The least significant difference (LSD) was calculated only when the ANOVA F-test was found to be significant at the *P*<0.05 probability level.
